# Next-Generation Metabolic Engineering of Capsaicinoids Biosynthesis in Chilli Pepper: Bridging Genomic Insights to Biotechnological Applications

**DOI:** 10.3390/biotech15030050

**Published:** 2026-07-01

**Authors:** Thumadath Palayullaparambil Ajeesh Krishna, Deepa Harikrishnan, Mathew Veena, Theivanayagam Maharajan, M. James, Minisha Udhayakumar, Parimala Gnana Soundari Arockiam Jeyasundar, Sherrie Jesulyn David, Ramar Dineshkumar, Reshma Rajan, Periyasamy Rathinapriya

**Affiliations:** 1 Division of Plant Science and Agricultural Biotechnology, School of Sciences, Bharata Mata College (Autonomous), Thrikkakara, Kochi 682 021, Kerala, India; 2 Department of Plant Biology and Plant Biotechnology, Quaid-E-Millath Government College for Women (Autonomous), Chennai 600 002, Tamil Nadu, India; deepa.hari1709@gmail.com; 3 Department of Botany, Sree Sankara College, Kalady, Ernakulam 683 574, Kerala, India; veena.mathew11@gmail.com; 4 Division of Plant Molecular Biology and Biotechnology, Department of Bioscience, Rajagiri College of Social Sciences, Kochi 683 104, Kerala, India; 5 ICAR — Indian Institute of Groundnut Research, Junagadh 362 015, Gujarat, India; james.m@icar.org.in; 6 Department of Biotechnology, Rathnavel Subramaniam (RVS) College of Arts and Science, Sulur, Coimbatore 641 402, Tamil Nadu, India; minishakumar7@gmail.com (M.U.); apgs_1987@yahoo.co.in (P.G.S.A.J.); 7 Department of Plant Biology & Plant Biotechnology, Women’s Christian College, Chennai 600 006, Tamil Nadu, India; sdavid@wcc.edu.in; 8 Department of Microbiology, Vivekanandha Arts and Science College for Women, Veerachipalayam, Sankagiri West, Salem 637 303, Tamil Nadu, India; dineshram625@gmail.com; 9 Department of Botany, St. Thomas College (Autonomous), Keerankulangara, Thrissur 680 001, Kerala, India; 20reshma15@gmail.com; 10 Horticultural and Herbal Crop Environment Division, National Institute of Horticultural and Herbal Science, Rural Development Administration, Wanju-gun 55365, Republic of Korea

**Keywords:** capsaicinoids, chilli peppers, genome editing, metabolic engineering

## Abstract

Chilli peppers (*Capsicum* species) have been widely used around the world because of their economic value and distinctive sensory characteristics. They contain abundant functional metabolites, especially a group of vanillylamide compounds belonging to the family of capsaicinoids, which have been exploited for medicinal, nutritional, agricultural, and cosmetic uses. The demand for capsaicinoid molecules is increasing day by day due to their high economic value and wide range of applications. Therefore, increasing bioactive metabolites, especially capsaicinoids in chilli peppers, is a major priority in the current scenario. Multi-omics approaches such as genomics, transcriptomics, proteomics, and metabolomics have substantially contributed to understanding the complex regulatory networks governing capsaicinoid biosynthesis. Key structural genes, transcription factors, and signaling pathways involved in the phenylpropanoid and branched-chain fatty acid pathways have been identified, providing valuable targets for metabolic engineering in chilli pepper. Despite these advances, the integration of genetic modification approaches for the targeted enhancement of capsaicinoid production remains limited in chilli pepper. Recent developments in biotechnology, particularly CRISPR/Cas-mediated genome-editing, enable the precise genetic modification of metabolic pathways and regulatory networks in plants. Therefore, it can contribute to the precise modification of key genes involved in the capsaicinoid biosynthesis pathway, offering potential strategies to enhance the capsaicinoid content in chilli pepper. However, CRISPR/Cas-mediated genome editing in chilli pepper is still in its early stages. There are currently no reports available on the successful enhancement of capsaicinoid content in chilli peppers through CRISPR/Cas-mediated genome editing. To date, no comprehensive review has evaluated the CRISPR-Cas-mediated genome-editing approaches for capsaicinoid metabolic engineering in chilli pepper. This review critically evaluates the recent advances in CRISPR/Cas–mediated metabolic engineering in chilli peppers, with particular emphasis on regulatory genes involved in capsaicinoid biosynthesis. Furthermore, multi-omics approaches are expected to complement these strategies by enabling the identification of key regulatory genes, the optimization of genome-editing targets, and the prediction of metabolic outcomes for enhanced capsaicinoid production. Overall, this review provides insights into improving capsaicinoid accumulation in chilli peppers through advanced genome-editing technologies.

## 1. Introduction

**Figure 1:**
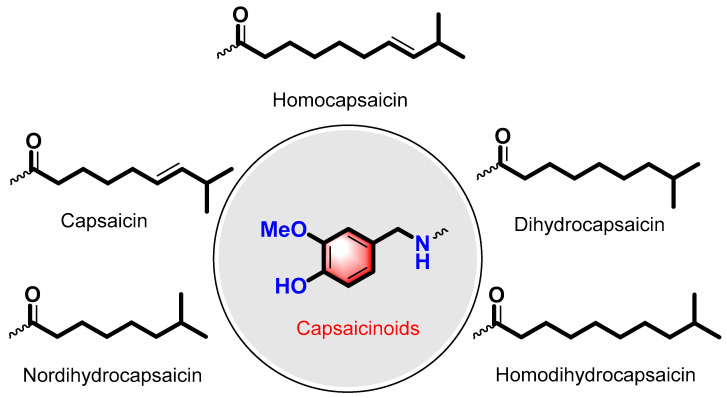
Major capsaicinoid molecules in chilli pepper. Capsaicinoids consist of a vanillyl (vanilloid) nucleus linked to an aliphatic chain via an amide bond. Structurally, capsaicinoids differ mainly in the composition of their fatty acidderived side chains. Variations in chain length and degree of unsaturation influence their hydrophobicity and pungent potency. This figure was generated using ChemDraw Professional 15.0.

*Capsicum* species (chilli peppers) are considered economically significant multi-purpose crops globally [1] due to their economic value, culinary uses, and medicinal properties in their fruit output [2,3,4,5,6]. Native to the tropical and temperate regions of the Americas, the genus includes various popular sweet and hot varieties, as well as wild types [7]. They belong to the family of Solanaceae, which comprise more than 30 species [8]. Of these, five are domesticated *Capsicum* species, including *C. annuum* L., *C. baccatum* L., *C. pubescens* Ruiz & Pav., *C. chinense* Jacq., and *C. frutescens* L. [9,10]. Among these, *C. chinense* Jacq. and *C. annuum* L. are the most widely cultivated species globally. The most distinctive characteristic of chilli peppers is their strong pungency, which is due to a group of vanillylamide compounds belonging to the capsaicinoids family. Capsaicinoids are a unique class of alkaloids found only in *Capsicum* fruits [11]. To date, 20 different capsaicinoid molecules have been identified across various *Capsicum* species [12,13,14]. These include major capsaicinoid molecules, such as capsaicin, dihydrocapsaicin, homocapsaicin, homodihydrocapsaicin, and nordihydrocapsaicin. Structurally, capsaicinoids vary only in the structure of their fatty acid chain composition (Figure 1). Capsaicin and dihydrocapsaicin are the major capsaicinoid found in chilli peppers and are primarily responsible for their characteristic pungency. Therefore, the commercial value of chilli peppers is largely determined by their capsaicinoid contents. Given their extensive pharmacological and industrial importance, improving capsaicinoid biosynthesis in chilli peppers has become a major focus of the current metabolic engineering research. The metabolic profile of chilli peppers varies depending on species, seasonality, environmental factors, and the plant’s life cycle [15,16,17]. As a result, selecting the most suitable chilli pepper species and cultivars for food, pharmaceutical, and agricultural applications can be challenging. Therefore, understanding the properties, composition, and factors influencing the metabolic profile of *Capsicum* fruits may help in selecting the appropriate cultivars and cultivation methods. Moreover, understanding the genome organization of *Capsicum* species provides insight into the genes/alleles involved in capsaicinoid biosynthesis. Therefore, the targeted modification of key genes/alleles through genome editing can enhance capsaicinoid accumulation via targeted metabolic engineering.

Currently, several promising approaches are available to enhance metabolic engineering in plants. The clustered regularly interspaced short palindromic repeat (CRISPR)-associated protein (Cas) system enables efficient genome editing in plants [18]. Due to its straightforward design, low cost, high efficiency, improved repeatability, and rapid breeding cycle, it is a powerful tool for metabolic engineering. The CRISPR-Cas system can be used to modify desirable traits of plants through knock-in or knock-out strategies. Although previous studies have independently addressed capsaicinoid biosynthesis and genome-editing technologies, the integrated evaluation linking capsaicinoid regulatory pathways with CRISPR-Cas-based precise engineering in *Capsicum* species remain limited.

In this comprehensive review, we examine the economic importance, nutritional and metabolic profiles, and health-promoting properties of chili peppers, with special emphasis on capsaicinoid biosynthesis and its genetic regulation. We also discuss the current applications, opportunities, challenges, and future prospects of metabolic engineering using the CRISPR-Cas approach to improve capsaicinoid production. The novelty of this review lies in providing insights into the integration of multi-omics data to guide CRISPR/Cas-mediated genome editing in chilli peppers, with a special focus on the regulatory genes involved in capsaicinoid biosynthesis. Overall, this review provides valuable insights into improved strategies for developing high-quality *Capsicum* cultivars with a high capsaicinoid content, which have significant practical application in pharmaceutical and nutritional fields. 

## 2. Economic Value and Current Production of Chilli Peppers

Vegetable crops are essential for food security [19], as they address the dietary needs of an expanding population [20] and help combat malnutrition [21]. Chilli peppers are widely valued across the world as vegetables, spices, and food additives [22], as well as for industrial applications in the pharmaceutical, ornamental, and cosmeceutical industries [23,24]. They are primarily used in food and pharmaceutical applications because they enhance flavor and add pungency to foods. Beyond their culinary usage, chilli peppers are regarded as nutritional powerhouses because they contain various vitamins, including vitamins A, E, C, and B complexes [10,25]; minerals, such as phosphorus, potassium, calcium, sodium, and iron [26,27,28]; and functional compounds, including flavonoids and capsaicinoids [25,29]. They have been widely used worldwide due to their valuable and distinctive economic characteristics, including color, pungency, and aroma, which are largely attributed to capsaicinoid accumulation. Chilli peppers comprise several economically and nutritionally important species, including *C. frutescens*, *C. baccatum*, *C. pubescens*, *C. chinense*, and *C. annuum*. Most importantly, they are valued for their distinctive flavors, varying degrees of pungency, and vibrant colors. Despite the wide variations in traits, *C. annuum* is the most commercially cultivated species [30,31] and is widely grown and consumed globally [3], with Asia being a major hub for both production and consumption. Remarkably, chilli peppers are cultivated in over 90 tropical and sub-tropical countries, covering 4.5 million hectares with an annual production of about 59 million tonnes. India is the leading producer of chilli peppers, contributing nearly 39.78% of global production, followed by China with 8.67% [32]. All varieties of chilli pepper are recognized as important cash crops for smallholder farming communities in developing countries [33]. Their production has increased in economic value since 1991, making them a vital component of international trade. Over the past 25 years, both fresh and dried chilli pepper production has increased six- and fourfold, respectively [30]. Recent data indicate that the global chilli pepper market is expanding rapidly, growing from USD 10.88 billion in 2025 to USD 11.56 billion in 2026. It is projected to grow at a compound annual growth rate (CAGR) of 6.63%, reaching USD 17.06 billion by 2032. This growth is driven by their high economic value and multipurpose applications in various industries. This rapidly expanding global demand emphasizes the urgent need for improved breeding and genetic modification strategies to develop high-yielding chilli varieties with an enhanced capsaicinoid content and improved commercial value.

## 3. Overview of Metabolites and Their Health Benefits of Chilli Peppers

**Figure 2:**
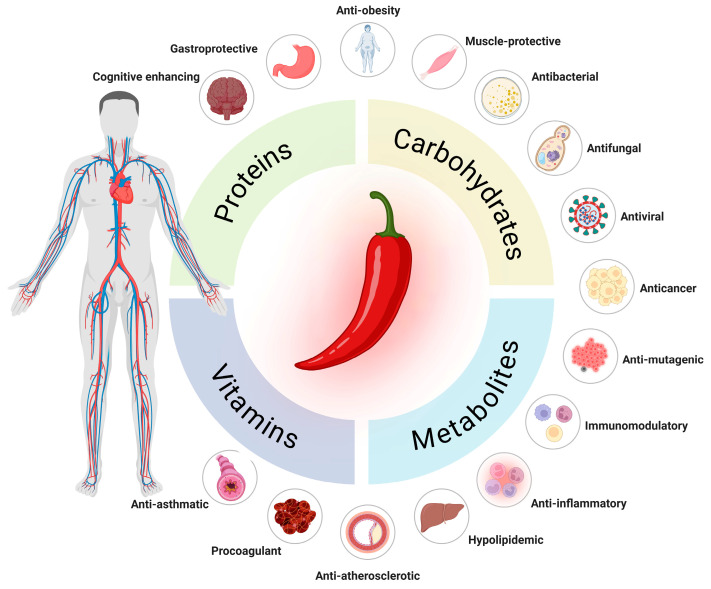
Various therapeutic applications of chilli peppers. Chilli peppers are rich sources of proteins, carbohydrates, vitamins, and diverse bioactive metabolites that contribute to their nutritional and medicinal value. The figure illustrates the health benefits of consuming chilli peppers, which are primarily attributed to the synergistic action of capsaicinoids, carotenoids, phenolic compounds, vitamins, and other phytochemicals present in chilli pepper. This image was created using BioRender: https://BioRender.com/kpp4oi7 (accessed on 20 February 2026).

Chilli peppers have gained increasing attention as functional foods due to their high nutritional values and diverse secondary metabolites, which provide various therapeutic applications (Figure 2). Nutritionally, chilli pepper fruits are naturally low in calories and fat, but contain high levels of essential minerals and vitamins [34]. Chilli peppers are a rich source of functional molecules due to their secondary metabolism [35,36]. These include flavonoids, carotenoids, capsaicinoids, and other naturally occurring bioactive compounds that contribute to their flavor, color, and pungency. Advances in nutritional biochemistry, metabolomics, and clinical nutrition have revealed that the *Capsicum*-derived compounds are involved in many biological processes, particularly in regulating energy balance, glucose metabolism, inflammation, oxidative stress, etc. [37]. Capsaicin and dihydrocapsaicin are the major capsaicinoids found in chilli peppers, constituting about 90% of total capsaicinoids. Capsaicin alone accounts for approximately 71% of total capsaicinoids in most pungent chilli varieties [38]. The remaining 10% consists of minor capsaicinoids that vary in the number of side-chain carbon atoms and due to unsaturations. Capsaicinoid compounds exert their biological effects mainly through the activation of the transient receptor potential vanilloid-1 (TRPV1) channel, a key mediator of sensory and metabolic responses [39,40,41]. This activation leads to increased intracellular calcium levels, the stimulation of catecholamine release, and the activation of the sympathetic nervous system [42]. As a result, thermogenesis, energy expenditure, and fat oxidation are enhanced, providing a mechanistic explanation for the anti-obesity effects associated with *Capsicum* species consumption [43,44]. In addition to TRPV1 activation, capsaicin also modulates the AMP-activated protein kinase (AMPK) pathway and peroxisome-proliferator-activated receptor alpha (PPARα), which regulate fat oxidation, improve insulin sensitivity, reduce body fat, and enhance liver and heart functions [45]. Capsaicin has been widely used as an analgesic for various types of arthritis [46,47]. Furthermore, it exhibits multiple pharmaceutical properties, including thermogenic, anti-inflammatory, antilithogenic, analgesic, and cardioprotective properties, and shows therapeutic potential in managing gastrointestinal disorders [48]. Therefore, the global demand for chilli peppers is increasing steadily due to their wide-ranging pharmaceutical applications. 

Additionally, chilli peppers are particularly rich in distinctive carotenoids, such as capsanthin and capsorubin. Nearly 50% of the total carotenoids are capsanthin, which has a structure consisting of 11 conjugated double bonds, a conjugated carbonyl group, and a 5-membered cyclopentane ring [49]. Owing to its extended conjugated double-bond system and keto functional groups, capsanthin exhibits strong antioxidant capacity [50,51], and is associated with chemopreventive, antitumor, skin photoprotective, anti-inflammatory, antidiabetic activities, etc. [51]. Apart from capsaicinoids and carotenoids, *Capsicum* fruits contain a diverse range of polyphenolic compounds. These include flavonoids such as luteolin and quercetin, as well as phenolic acids like ferulic and caffeic acids. Together, these compounds enhance antioxidant defences [52] and help modulate inflammatory and metabolic signalling pathways [53]. Collectively, these findings highlight the considerable therapeutic and industrial importance of capsaicinoids, particularly in pharmaceutical, nutritional, and functional food applications. However, current capsaicinoid production relies primarily on extraction from chilli peppers, which is often limited by low metabolic yields and environmental variability. These limitations have intensified interest in understanding the molecular mechanisms governing capsaicinoid biosynthesis and regulation. In particular, elucidating the key genes and regulatory networks involved in this pathway is essential for targeted metabolic engineering. Such knowledge provides the foundation for precise genomic editing interventions aimed at improving capsaicinoid accumulation and generating high-quality chilli pepper cultivars with superior bioactive compound production.

## 4. Next-Generation Metabolic Engineering in the Post-Genomic Era

**Figure 3:**
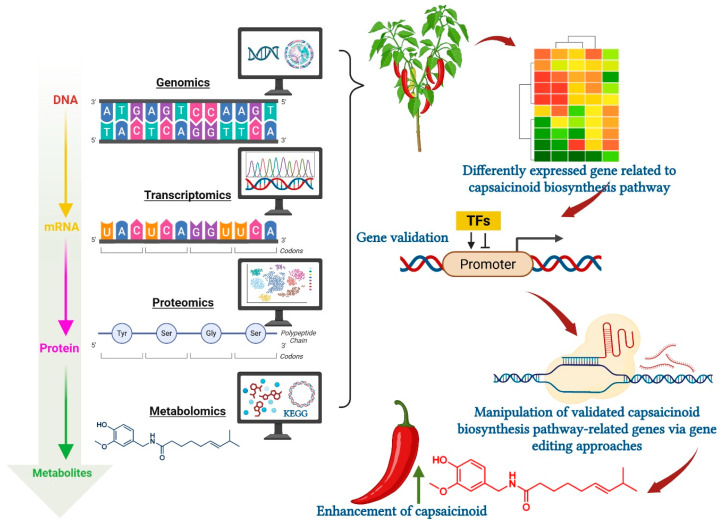
Multi-omics approaches for metabolic engineering in chilli pepper. Multi-omics approaches, including genomics, transcriptomics, proteomics, and metabolomics, are employed to comprehensively investigate the molecular mechanisms underlying capsaicinoid biosynthesis pathway. These multi-omics datasets are used to guide precise CRISPR/Cas-mediated metabolic engineering in chilli pepper. This integrated strategy facilitates the development of chili pepper cultivars with enhanced capsaicinoid content. This image was created using BioRender: https://BioRender.com/kpp4oi7 (accessed on 20 February 2026).

The manipulation of cellular processes is one of the most effective strategies to enhance the biosynthesis of desirable plant metabolites [54,55]. Therefore, substantial research efforts are required to identify genetic factors, rigorously quantify the metabolic phenotype, and understand the kinetic control in metabolic networks. In the post-genomic era, multi-omics approaches, such as genomics, transcriptomics, proteomics, and metabolomics, have significantly advanced the field of metabolic engineering. Many studies have highlighted the application of multi-omics approaches in plant metabolic engineering in plants [56,57,58]. The integration of these approaches has provided novel insights into gene function, enabling a better understanding of the complexity of plant-specialized metabolite pathways in chilli peppers, particularly for capsaicinoid biosynthesis (Figure 3). The identification and characterization of both positively and negatively regulated genes involved in capsaicinoid biosynthesis can support the enhancement in the capsaicinoid content in chilli peppers through genome-editing approaches. Genomics plays an important role in understanding the structural and functional dynamics of plant genomes [56], and it facilitates the identification of key genes involved in metabolic pathways [59]. To date, many studies have successfully mapped the genome organization of both cultivated and wild chilli peppers [60,61,62,63,64,65]. These studies have provided valuable data on the evolutionary divergence between wild progenitors and cultivated varieties, as well as trait-specific variations in chilli peppers. We summarize the comparative features of chilli pepper genome sequences in Table 1. Recently, Wahyuni and co-workers [66] identified *C. annuum* Long Sweet (Accession No: CGN23289) and *C. chinense* AC2212 (Accession No: CGN21469) as promising accessions with higher contents of several health-associated metabolites [66]. Therefore, understanding the genome organization of these chilli pepper accessions may help identify key genes associated with an enhanced capsaicinoid accumulation. This highlights the need to focus on the structural and functional genomics of chilli pepper cultivars with a high capsaicinoid content. Currently, whole-genome shotgun sequencing and resequencing datasets of chilli peppers are available in public databases. These high-quality genome sequences provide a valuable platform for improving the nutritional, medicinal, and horticultural value of chilli pepper and for developing improved varieties [61,65,67]. In particular, annotated genome sequences enable comparative genome studies and facilitate the identification of genes involved in capsaicinoid biosynthesis. Genomic approaches, including genetic analysis, genome mining, genomic selection, or genome-wide association studies, have significantly contributed to deciphering the biosynthetic pathways of metabolites. Overall, genomic information and advanced biotechnological tools provides opportunities to design targeted cellular modifications and enhance the production of desirable metabolites in chilli peppers.

Similarly, the transcriptomic sequencing datasets have emerged as invaluable genomic resources for reconstructing gene expression profiles, providing valuable insights into gene regulatory mechanisms [68,69,70]. Especially, RNA-sequencing platforms enable the decoding of genes and regulatory networks that govern plant metabolite biosynthesis [71,72]. For example, Wahyuni and co-workers [66] analyzed the morphological traits and metabolite contents across 32 *Capsicum* species accessions. This study revealed that major metabolites, including the carotenoid, flavonoid glycoside, capsaicinoid, and vitamin (C & E) content in the fruits, differed significantly between accessions. It indicates that the genetic variations among accessions contributes to the greater variation in metabolic content of the chilli pepper fruits [66]. Therefore, transcriptomic data combined with Kyoto Encyclopedia of Genes and Genomes (KEGG) pathway analysis have enabled the identification of key genes involved in the biosynthesis of metabolites in chilli peppers. By integrating RNA-seq data with metabolic profiling, researchers can identify differentially expressed genes (DEGs) associated with capsaicinoid accumulation. For example, Liu and co-workers [73] identified 54,045 DEGs in the placenta and pericarp tissues of *C. frutescens* L. through de novo transcriptome analysis. They also reported three structural genes, such as *dihydroxyacid dehydratase* (*DHAD*), *prephenate aminotransferase* (*PAT*), and *threonine deaminase* (*TD*), involved in the biosynthesis pathway of capsaicinoids [73]. Furthermore, Zhang and co-workers [74] identified 20 key genes associated with capsaicin biosynthesis and accumulation through transcriptome analysis. To date, several studies have successfully identified candidate genes involved in the biosynthesis of capsaicinoids using transcriptomic analysis (Table 2). However, despite the considerable progress in identifying potential genes based on the transcriptome, the functional characterization of key biosynthetic genes and transcription factors (TFs) regulating capsaicinoid biosynthesis remains limited.

**Table 1:** Comparison of genome sequence data of various chilli peppers. The details such as the name of the chilli pepper, the platform used for genome sequencing, genome size, and total genome length, sequencing depth, number of scaffold, transposable elements, and total number of genes are provided with the respective citation.

*Capsicum* Species	Platform Used	Genome Size (Gb)	Total Sequence Length (Gb)	Sequencing Depth (X)	Number of Scaffold	Transposable Elements (%)	Genes Number	Ref.
*C. annuum* (CM334)	Genome Analyzer IIx and HiSeq 2000	3.48	650.2	186.6	37,989	76.4	34,903	[65]
*C. chinense* (PI159236)	Genome Analyzer IIx and HiSeq 2000	3.14	289.6	83.2	239,495	79.6	33,788	[65]
*C. annuum* (Zunla-1)	Illumina Genome Analyzer II	3.26	477.37	146.43	28,149	80.9	35,336	[60]
*C. annuum* (Chiltepin)	Illumina Genome Analyzer II	3.07	295.85	96.37	30,293	81.4	34,476	[60]
*C. baccatum* (PBC81)	Illumina Hiseq 2500	3.9	526.7	136.1	2083	85.0	35,874	[75]
*C. chinense* PI159236	Illumina Hiseq 2500	3.2	425.7	132.2	1557	85.0	35,009	[75]
*C. annuum* (CM334 F_1_)	Illumina HiSeq X Ten sequencer	3.2	104.7	56	83,391	-	-	[76]

Proteomics helps identify proteins involved in metabolic pathways [77], thereby providing insights into regulatory proteins and transcription factors that are directly involved in the biosynthetic pathways of desirable metabolites [78]. The initial progress on a proteomics study in chilli peppers identified the key protein or enzyme involved in the biosynthesis pathway of ethylene [79], capsanthin, tocopherols, and carotenoids [80]. For example, Liu and co-workers [80] identified 23,349 transcripts and 5455 protein groups at various fruit developmental stages in two varieties of *C. annuum* L. (SJ11-3′ and 06g19-1-1-1) using RNA-sequencing and label-free quantitation technologies. The key findings of this study showed that the synthesis of capsanthin is mainly regulated by geranylgeranyl phosphate synthase 1 (GGPS1) and capsanthin capsorubin synthase 1 (CCS1), and the synthesis of tocopherol is regulated by GGPS1, geranylgeranyl diphosphate reductase (GGR), and tocopherol cyclase (TC). Moreover, the GGPS1 is also involved in the regulation of tocopherol synthesis [80]. This study provides insight that the integration of transcriptomics and proteomics data uncovered a number of candidate transcripts/proteins that might be involved in capsanthin, tocopherol, and ascorbate biosynthesis in chilli pepper. However, limited proteomic information is currently available regarding the biosynthesis of capsaicinoids in chilli peppers. Recently, Momo and co-workers [81] integrated a multi-omics approach to identify proteins involved in fruit development and metabolite biosynthesis in the placenta and pericarp tissues of two *Capsicum* species such as *C. chinense* and *C. annuum*. This study identified 4473 and 2012 proteins from the pericarp and placenta tissues, respectively. Key proteins related to capsaicinoid biosynthesis, such as acyltransferase 3 (AT3), 3-oxoacyl-[acyl-carrier protein (ACP)] synthase (KAS), 4-coumaroyl co-A ligase (4CL), and 3-ketoacyl-coA synthase 3 (KCS3), were identified in the placenta of *C. chinense* (highly pungent variety), along with J-domain proteins and TFs such as bHLH112, MYB101, NAC, MYB 14-like, and Cyt p450 CYP82D47, suggesting their role in capsaicinoid and secondary metabolite biosynthesis in chilli pepper [81]. These findings provide valuable multi-omics resources for future functional studies and support the development of improved chilli pepper varieties with a higher capsaicinoid content through genome-editing approaches.

**Table 2:** Insight into the key genes involved in the capsaicinoid biosynthesis pathway in *Capsicum* species through transcriptome analysis. This table listed information about the name of the *Capsicum* variety, the type of tissue used for sequencing, the sequencing platform, and the identified key DEGs related to the capsaicinoid biosynthesis.

*Capsicum* Species	Name of the Variety	Platform Used	Tissue Used	No. of DEGs Identified	Identified Key DEGs Involved in the Biosynthetic Pathway of Capsaicinoid	Ref.
*C. frutescens* L.	Xiaomila	Illumina HiSeq 2000	Placenta and pericarp	54,045	*DHAD*, *TD*, *PAT*	[73]
*C. frutescens* L.	Guijiangwang	Illumina HiSeq 2000	Placenta	28,434	*PAL*, *C4H*, *ACS*, *NADH-GOGAT*, *BCKDH*, and *AT*	[74]
*C. chinense* Jacq.	Shuanla	Illumina NovaSeq 6000	Fruits	15,741	*PAL*, *C4H*, *4CL*, *HCT*, *C3H*, *COMT*, and *PAMT*	[82]
*C. baccatum* L.	Cultivar-JC	Illumina HsSeq-2000	Fruits	4835	*4CL*, *CHS*, and *COMT*	[83]
*C. frutescens* L.	Cultivar-YB	Illumina HsSeq-2000	Fruits	3408	*4CL*, *CHS*, and *COMT*	[83]
*C. annuum* L. var. *glabriusculum*	*Chiltepin*	Illumina HiSeq 2500	Fruits	5217	Detected 412 DEGs associated with the capsaicinoid accumulation	[84]

## 5. Biosynthesis Pathway of Capsaicinoids in Chilli Pepper

Capsaicinoids are phenolic alkaloid molecules synthesized in the *Capsicum* fruits, which mainly include capsaicin and dihydrocapsaicin. Their biosynthesis occurs in placental tissue between 16 days post-anthesis and the mature green stage, and is regulated through the coordinated activity of the branched-chain fatty acid and phenylpropanoid (PP) pathways. The latter one generates vanillylamine, while the first pathway produces precursor fatty acids with 9 to 11 carbon atoms, which are then condensed into capsaicinoid synthesis through enzymatic catalysis reactions [85,86]. The catalytic enzyme phenylalanine ammonia-lyase (PAL) facilitates the conversion of L-phenylalanine to trans-cinnamate. Subsequently, cinnamate 4-hydroxylases (C4Hs) convert the trans-cinnamic acid into 4-coumaric acid. The formed 4-coumarate is subsequently activated by its conversion into 4-coumaroyl-CoA via the action of the enzyme 4CL. This 4-coumaroyl CoA is further converted into 4-coumaroyl shikimate by the action of the hydroxycinnamoyl-CoA:shikimate hydroxycinnamoyl transferase (HCT) enzyme [82]. Then, the 4-coumaroyl shikimate is hydroxylated, yielding caffeoyl shikimate, a process catalyzed by the enzyme 4-coumarate 3-hydroxylase (C3H), a cytochrome P450-dependent mono-oxygenase. Caffeoyl shikimate is converted to caffeoyl CoA via HCT. A specialized C3H enzyme is reported to convert 4-coumaroyl CoA into caffeoyl CoA, requiring zinc and ascorbate for its activity [87]. Caffeoyl-CoA thus formed is transformed into feruloyl-CoA by the catalysis of the Caffeoyl-CoA O-methyltransferase (CCOAOMT) enzyme, which methylates the 3′-hydroxyl position of caffeoyl-CoA, utilizing S-adenosyl-L-methionine (SAM) as the methyl donor [88]. Feruloyl-CoA is transformed into vanillin, the rate-limiting step in capsaicinoid biosynthesis, facilitated by hydroxycinnamoyl-CoA hydratase/lyase (HCHL) [89]. The conversion of vanillin to vanillylamine is considered a very crucial step in the biosynthesis of capsaicinoids. This reaction is a transamination process catalyzed by vanillin aminotransferase (VAMT). Gamma-aminobutyric acid (GABA) acts as the amino donor, while pyridoxal phosphate (PLP) remains as a coenzyme in this reaction. The vanillylamine subsequently reacts with the branched-chain fatty acid to produce capsaicin [90].

**Figure 4:**
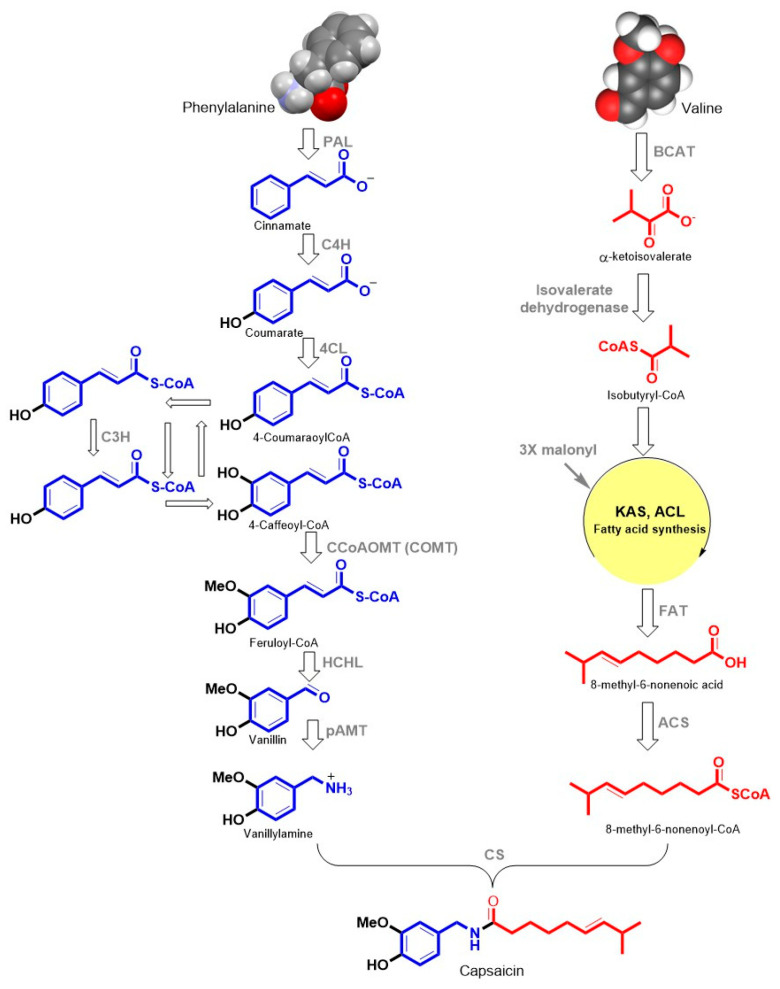
Biosynthesis pathway of capsaicinoid molecules in chilli pepper. This schematic diagram showed the capsaicin biosynthesis pathway and their regulatory genes. This image was created in ChemDraw Professional 15.0.

The initial step in the branched-chain fatty acid (BCFA) pathway involves the transformation of valine into α-ketoisovalerate, facilitated by the branched-chain amino acid transaminase (BCAT) enzyme [91]. The formed alpha-ketoisovalerate is converted to isobutyryl-CoA by the enzyme branched-chain alpha-ketoacid dehydrogenase/decarboxylase (BCKDH) complex, a critical step in the BCFA pathway [92]. The subsequent extension and modification of the resulting fatty acid chain is driven by the combined action of ketoacyl-ACP synthase (KAS) and acyl carrier protein (ACP). The conversion occurs to form 8-methyl-6-nonenoic acid through acyl-ACP thioesterase (FatA), which then subsequently transformed into 8-methyl-6-nonenoyl-CoA via acyl-CoA synthetase (ACS) [89]. As mentioned earlier, the 8-methyl-6-nonenoyl-CoA formed by the BCFA and the earlier mentioned vanillylamine formed from the phenylpropanoid pathway undergo a condensation reaction to form capsaicin catalyzed by the enzyme capsaicin synthase (CS), which is encoded by the *Pun1* gene, or *AT3*. Although the capsaicinoid biosynthesis pathway is well-described (Figure 4) [93,94,95], nevertheless, a few regulatory factors/genes have been identified and/or functionally characterized so far. In particular, TFs regulating PP flux, BCFA precursor, and *pun1* expressions remain poorly underexplored. The identification of these regulatory nodes is critical for targeted genome-editing and metabolic engineering strategies. The overall capsaicinoid accumulation relies not only on the structural enzyme activity but equally on the precursor availability, subcellular compartmentalization, and metabolic flux between competing pathways. Therefore, the precise manipulation of these pathway fluxes represents a promising strategy to enhance the capsaicinoid yield in *Capsicum* species.

## 6. Overview of Key Genes Involved in Biosynthesis Pathway of Capsaicinoids

Generally, both genetic and environmental factors significantly influence capsaicinoid accumulation in chilli peppers, with allelic variations representing a major determinant of the differences in pungency among cultivars [17,96,97]. These variations are largely driven by the differential expression of genes regulating capsaicinoid biosynthesis, signal transduction, and enzyme activity. Thus far, many capsaicinoid-biosynthesis-involved genes have been identified, and those genes encode enzymes such as capsaicin synthase (CS), phenylalanine ammonia-lyase (PAL), branched-chain amino acid transferase (BCAT), cinnamic acid 4-hydroxylase (C4H), acyl-ACP thioesterase/transferase (ACL), aminotransferase (AT3), 4-coumarate-CoA ligase (4CL), 3-ketoacyl-ACP synthase (KAS), acyl-ACP thioesterase (FatA), acyl-CoA synthetase (ACS), etc. [98,99,100]. Earlier studies showed that genes such as *pAMT*, *KAS*, and *AT* are tissue-specific genes which are expressed in the placental tissue and are involved in capsaicin synthesis in chilli peppers [74]. Furthermore, capsaicin and dihydrocapsaicin compounds are synthesized from the glandular epidermal cells into the subcuticular canals, which enlarge to form blisters along the epidermis. The blister formation on the placenta of chilli peppers requires the *Pun1* gene [101], which correlates with the higher accumulation of capsaicinoids [91]. Moreover, the *Pun1* gene encodes the AT3 enzyme indispensable for the biosynthesis of capsaicinoids [93], which corresponds to the condensation of vanillylamine and fatty acids in the last stage of capsaicinoid biosynthesis [102]. The *Pun1* (*AT3*) gene is recognized as a key determinant of pungency, as mutations or deletions in this locus result in non-pungent phenotypes due to the disrupted capsaicinoid synthesis. Previous studies revealed that silencing the *AT3* negatively influences the expression of other important genes like *pAMT*, *BCAT*, *KAS*, and *ACL*, suggesting that *AT3* (*Pun1*) also functions as a key regulator in the transcription of these biosynthetic genes [103]. Furthermore, many researchers have highlighted the key genes involved in the biosynthetic pathway of capsaicinoids in chilli pepper [95]. Understanding the key genes associated with the biosynthesis pathway of capsaicinoids (Figure 4) and their proper utilization is crucial for enhancing their production in chilli peppers.

## 7. Opportunity and Challenges of CRISPR/Cas-Mediated Metabolic Engineering in Chilli Pepper

Genome editing is a powerful genetic engineering approach that equips precise modifications at specific genomic loci [104]. It includes meganucleases, zinc-finger nucleases (ZFNs), transcription activator-like effector nucleases (TALENs), and CRISPR/Cas techniques, which permit the insertion, deletion, or modification of DNA segments by creating targeted breaks and leveraging cell repair pathways (nonhomologous end joining or homology-directed repair) for modifications [105,106]. Many researchers have highlighted an overview of their functions and mechanism [107,108] as well as their applications in crop improvements. Many intriguing investigation outbreaks have been seen in the CRISPR/Cas9 tool employed to alter the metabolic profiles in many plants [109,110,111,112]. It enables targeted, site-specific modifications in native genomes without the inclusion of foreign DNA, thereby overcoming many regulatory obstacles and allays societal worries regarding transgenics. The CRISPR/Cas9 variants, such as CRISPR activation (CRISPRa) and CRISPR interference (CRISPRi), are reversible tools for the transcriptional regulation of targeted genes [113,114,115]. In the CRISPRa variant, nuclease-dead Cas9 (dCas9) shall be combined with transcription activators to bring in RNA polymerase and upregulate gene expression. But the CRISPRi variant, the dCas9 system, inhibits RNA polymerase’s initial binding and downregulates the gene expression, interfering with the transcription of targeted genes. Therefore, these CRISPR/Cas variants could help modulate metabolic pathways by either upregulating or downregulating the target genes in the metabolic pathway, thereby enhancing metabolite accumulation without breaking the genomic DNA. Therefore, the CRISPR/Cas tool is the most promising approach for metabolic engineering, especially capsaicinoids in chilli peppers. Increasing the desirable content of capsaicinoids in chilli peppers can be achieved by knocking in or out specific genes that act as positive or negative regulators in the biosynthesis pathway of capsaicinoids.

The CRISPR/Cas system has been successfully used to alter metabolic pathways in several plant species such as *Atropa belladonna* [116], *Symphytum officinale* [117], *Solanum lycopersicum* [118], *Dioscorea zingiberensis* [119], *Cichorium intybus* [88], *Glycine max* [120], *Papaver somniferum* [121], *Oryza sativa* [122], and *Medicago truncatula* [123]. For example, γ-aminobutyric acid (GABA) is a four-carbon nonprotein amino acid and has recently received considerable attention as a health-promoting functional metabolite [124]. Functionally, the *GABA-TP1*, *GABA-TP2*, *GABA-TP3*, *CAT9,* and *SSADH* genes are involved in GABA metabolism in *Solanum lycopersicum*. The multiplex CRISPR/Cas9-mediated mutagenesis of these genes (single or double or triple or quadruple) showed a significantly increased GABA content in the leaves and fruits in edited lines [118]. On the other hand, the pyrrolizidine is a toxic alkaloid compound found in certain plant species; there are many secondary pathways causing the pyrrolizidine contamination of other plants, including medicinal and food-based plants, which pose a risk of human intoxication [125]. The homospermidine synthase (HSS) has evolved from deoxyhypusine synthase (DHS) and catalyzes the first step in the pyrrolizidine biosynthesis pathway. The CRISPR/Cas9-mediated knockout of the *HSS* gene leads to a reduction in the pyrrolizidine alkaloid level in the mutated lines of *Symphytum officinale* [117]. The use of CRISPR/Cas9-mediated genome editing in *Capsicum* species is still in its early stages (Table 3). Thus far, there is no information available on the utilization of CRISPR/Cas-mediated genome editing for enhancing capsaicinoid biosynthesis in *Capsicum* species. Previously, Bulle and co-workers [126] developed the CRISPR/Cas9-mediated gene delivery system in *C. annuum,* targeting the *phytoene desaturase* (*PDS*) gene. This protocol achieves an up to 62.5% editing efficiency by using the biolistic delivery of CRISPR/Cas9 reagents [126]. Therefore, this protocol can be used to alter the genes related to the capsaicinoid biosynthesis pathway and increase the desirable capsaicinoid content in chilli pepper.

**Figure 5:**
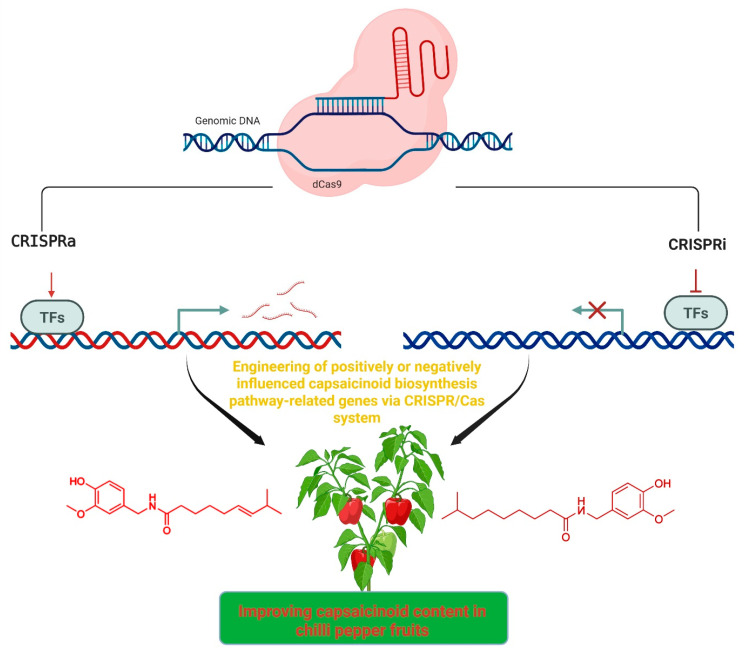
Application of CRISPR/Cas variants for improving capsaicinoid contents in chilli pepper. Catalytically inactive Cas9 (dCas9)-based CRISPR activation (CRISPRa) and CRISPR interference (CRISPRi) systems are employed to modulate the expression of genes involved in the capsaicinoid biosynthetic pathway. In the CRISPRa approach, dCas9-associated transcriptional activators enhance the expression of target genes by promoting TF, thereby increasing transcript accumulation and stimulating capsaicinoid biosynthesis. Conversely, CRISPRi represses gene expression by preventing TF binding or transcriptional initiation, resulting in reduced expression of target genes. The targeted activation or repression of positive and negative regulators of the capsaicinoid biosynthetic pathway enables precise metabolic engineering for optimizing capsaicinoid production and improving pungency-related traits in chili pepper. Created in BioRender: https://BioRender.com/kpp4oi7 (accessed on 20 February 2026).

The TFs are important components involved in the transcriptional regulation of genes. The manipulation of certain TFs leads to transcriptomic changes in the expression of specific target genes in metabolic pathways, which leads to enhanced desirable metabolites [100]. Therefore, the transcriptional regulation of capsaicinoid-biosynthesis-pathway-related genes could be beneficial for enhancing their production (Table 4). However, the application of the CRISPR/Cas tool for enhancing capsaicinoid biosynthesis in *Capsicum* species remains in its early stages. Previously, some interesting research progress has been seen in the manipulation of the capsaicinoid profiles in chilli peppers. Most of the studies showed that the virus-induced gene silencing (VIGS) technology was used to alter the capsaicinoid biosynthesis pathway in chilli peppers. For example, Yu and co-workers [130] revealed that the CcMYB24 TF acts as a negative regulator of the PP metabolic pathway. The silencing of the *CcMYB24* gene generally enhances the expression levels of capsaicin biosynthesis genes like *COMT*, *CO*, and *AT3* in the placenta of *Capsicum chinense* Jacq. (Hainan Huangdenglong pepper); consequently, the contents of capsaicin increased by more than 31.72% [130]. Furthermore, phenylalanine is an important starting material of the PP pathway that is shared by the biosynthesis of lignin and capsaicin. The key targets for increasing capsaicinoids involve reducing the flow of precursors into the lignin pathway to increase the availability of raw materials for capsaicin synthesis, thus downregulating the lignin pathway helps boost the production of capsaicin. The cinnamoyl-CoA reductase (CCR) or cinnamyl alcohol dehydrogenase (CAD) is an important enzyme in the PP pathway that leads to lignin synthesis [130,131,132]. Wu and co-workers [131] revealed that silencing the *CCR1* gene leads to reduced lignin levels by 10.2% in the pericarp and 13.5% in the placenta of *C. chinense*, which then caused the capsaicin levels to increase by 5% and 9.6%, respectively. Similarly, the levels of lignin in the pericarp and placenta of the *CcCCR2*-silenced *C. chinense* decreased by 18.8% in the pericarp and 22.8% in the placenta, which then caused the capsaicin levels to increase by 16.2% and 19.1%, respectively [131]. These studies offer a comprehensive understanding of increasing the capsaicinoid content in chilli peppers via the knocking in or out of specific genes that act as positive or negative regulators of the capsaicinoid biosynthesis pathway (Figure 5) via CRISPR/Cas-mediated genome editing. However, it is necessary to identify more TFs and their roles associated with the biosynthesis of capsaicinoids in chilli pepper.

The CRISPR/Cas tool is an efficient and easy-to-operate gene knocking-in or -out system widely used for metabolic engineering in plants. General bottlenecks in CRISPR/Cas include a low transformation efficiency, genotype-dependent regeneration capacity, and tissue culture recalcitrance, which limit its application on a large scale. For example, Park and co-workers [138] investigated which strains of *Agrobacterium tumefaciens* (AGL1, EHA101, and GV3101) are better for CRISPR/Cas-mediated genome editing in chilli pepper cultivars (hot pepper cultivar CM334 and bell pepper cultivar Dempsey). This study revealed that the *Agrobacterium tumefaciens* strain *EHA101* exhibited the highest editing efficiency for the *CaMLO2* gene in both cultivars Dempsey and CM334 among the tested strains [138]. Moreover, this study revealed that *Agrobacterium tumefaciens* strain GV3101 showed better callus-inducing activity in bell pepper cultivar Dempsey. Furthermore, this study suggests that the callus induction rate by an *Agrobacterium tumefaciens* strain can differ depending on the chilli pepper cultivars [138]. Therefore, research attention should be given to improving CRISPR/Cas genome editing in chilli peppers, particularly in how to deliver RNP complexes for better safety and effectiveness. Sometimes, the genetic alterations at off-target sites are a primary challenge in CRISPR-Cas technology. To ensure the integrity and biosafety of edited lines, rigorous in silico guide RNA (gRNA) design and validation are essential. Nowadays, deep- and machine-learning tools based on artificial intelligence provide excellent data processing and analytical capabilities, which offer efficiency for CRISPR/Cas-based genome-editing strategies [139,140]. Overall, the artificial intelligence tools allow detecting off-target genes, optimizing editing processes, designing gRNA, and predicting genome-editing outcomes [141]. Moreover, the integration of artificial intelligence tools could help with the development and improvement of sophisticated genome-editing methods like base editing, prime editing, and epigenome editing.

Furthermore, the main obstacles to CRISPR/Cas-mediated genome editing in chilli peppers are the difficulty in delivering the reagents and the plant regeneration protocol, both of which are highly dependent on the genotype and variety. Recently, Bulle and co-workers [126] optimized the CRISPR/Cas9-mediated gene delivery system in *C. annuum,* targeting the *PDS* gene. This protocol achieves an up to 62.5% editing efficiency by using CaMV 35S-driven Cas9, dual-sgRNA, and the biolistic delivery of CRISPR/Cas9 reagents, producing stable, heritable, and often non-genetically modified mutants with visible albino phenotypes for selection [126]. Moreover, previous studies showed that successful CRISPR/Cas9-mediated gene editing depends on better delivery systems. The CRISPR/Cas reagent delivery methods such as biolistic, polyethylene glycol (PEG), and *Agrobacterium*-mediated transformation have been reported in chilli pepper. Among these methods, *Agrobacterium*-mediated transformation showed a higher transformation efficiency than other methods [126,127,128]. Interestingly, it depends upon requiring a highly compatible combination of *Agrobacterium* strains, vector design, and host tissue (explants) regeneration efficiency. An optimized CRISPR/Cas-mediated gene delivery system is crucial for successfully modifying capsaicinoid-biosynthesis-pathway-related genes, which could help enhance the capsaicinoid. Therefore, efficient *Agrobacterium*-mediated transformation, protoplast transfection, and the direct delivery of ribonucleoprotein (RNP)-based editing techniques can help easily generate transgene-free edited plants. We hope that the CRISPR/Cas genome-editing era will be a landmark in the development of improved varieties of chilli peppers with a higher capsaicinoid content in the future (Figure 5). It may provide a sufficient quantity of capsaicinoid molecules in the food, agriculture, and pharmaceutical industries as raw materials for many value-added products. Therefore, scientists should emphasize the future focus on developing an efficient CRISPR/Cas-mediated genome-editing protocol in chilli pepper.

## 8. Conclusions and Future Prospects

Plants are capable of producing a diverse array of secondary metabolites that are essential for their fundamental functions, including growth, development, reproduction, defence, and adaptability. Most of the plant secondary metabolites have biological proper-ties and are beneficial for human beings as nutraceuticals and pharmaceuticals. Chilli peppers are an excellent source of bioactive molecules, especially a group of vanillylamides belonging to the family of capsaicinoids, which are harnessed for their therapeutic, aesthetic, and dietary values. The demand for chilli-pepper-derived capsaicinoids is increasing day by day owing to their diverse applications. The capsaicinoid content of chilli peppers varies due to species, seasonality, environmental factors, and even the plant’s life cycle. Nowadays, scientists are trying to meet the demand for capsaicinoids through various strategies. Synthetic capsaicinoids, like nonivamide, are widely used as alternatives to natural ones in various industries, including self-defence sprays, pesticides, and analgesic creams, due to their lower production costs and higher consistent purity. However, their use comes with several significant drawbacks, ranging from health risks to environmental impact. Understanding the dominant genetic drivers of capsaicinoid biosynthesis is crucial for rendering precise metabolic engineering and improving the quality of chilli peppers.

In the post-genome era, genomics, transcriptomics, proteomics, and metabolomics represent an integrated, multi-omics approach focusing on functional biology that helps to understand the genome’s organization and the key genes responsible for metabolic pathways in chilli peppers. Therefore, scientists need to focus more on the functional mechanisms of capsaicinoid biosynthesis in chilli peppers. The CRISPR/Cas system provides a promising platform for the targeted metabolic engineering of capsaicinoid biosynthesis in *Capsicum* species. Furthermore, the integration of genomics, transcriptomics, proteomics, metabolomics, and artificial-intelligence-assisted analytical tools has considerably improved our understanding of the regulatory networks governing capsaicinoid accumulation. These advances offer new opportunities for identifying candidate genes, optimizing genome-editing targets, predicting editing outcomes, and improving the precision of metabolic engineering strategies. However, despite these advances, the practical implementation of CRISPR/Cas-mediated metabolic engineering in *Capsicum* species remains limited.

Major challenges include the low transformation efficiency, genotype-dependent regeneration capacity, tissue-culture recalcitrance, and lack of robust and universally applicable gene-delivery systems. In addition, the potential off-target modifications and unintended metabolic consequences require a thorough assessment to ensure the genetic stability and biosafety of the edited plants. The regulatory status of genome-edited crops also varies considerably among countries, which may influence the commercialization and adoption of edited chilli pepper cultivars. Moreover, most studies conducted to date have been performed under controlled laboratory or greenhouse conditions, whereas a comprehensive validation of the edited lines under field environments is still lacking. Therefore, future research should prioritize the development of efficient transformation and regeneration protocols, improved delivery systems for genome-editing reagents, rigorous off-target evaluation frameworks, and the large-scale field validation of edited germplasm. Although the integration of artificial intelligence, multi-omics approaches, and CRISPR/Cas technologies represents a promising direction for capsaicinoid metabolic engineering, significant technical, biological, and regulatory challenges must be addressed before these approaches can be routinely applied in practical *Capsicum* breeding programmes. Addressing these limitations will be essential for translating the current genomic knowledge into commercially viable, high-capsaicinoid chilli pepper cultivars that perform stably under diverse environmental conditions.

## Figures and Tables

**Table 3:** Overview of CRISPR/Cas-mediated genome editing in chilli pepper. Details on target gene, gene function, CRISPR/Cas editing type, method of construct delivery, vector, and promoter were included with reference.

*Capsicum* Species	Targeted Gene	Gene Function	Editing Type	Method of Delivery	Vector	Promoter	Efficiency (%)	Ref.
*C. annuum* L.	*PDS*	Carotenoid biosynthesis	Mutagenesis	Biolistic	*pKSE401*	CaMV 35S	62.5	[126]
*C. annuum* L.	*ERF28*	Anthracnose resistance	Mutagenesis	*Agrobacterium*	*pTX171*	CaMV 35S	70.37–74.28	[127]
*C. annuum* L.	*PAD*	Inducing parthenocarpy	Mutagenesis	PEG	-	-	7.2–11.3	[128]
*C. annuum* L.	*MLO2*	Powdery mildew resistance	Mutagenesis	PEG	-	-	6.3–17.7	[129]

**Table 4:** Transcription factors (TFs) and their roles in regulations of capsaicinoid-biosynthesis-pathway-related genes. Detail of name of TFs and their targeted genes and roles, critical regulatory elements (CREs), and key findings are provided with reference.

TF	Effects	Target Gene	CREs	Key Findings	Ref.
CcMYB4	Repressor	*4CL*	CACCCA	CcMYB4 binds to the *Cc4CL* promoter and inhibits its expression, thereby regulating capsaicinoid biosynthesis	[82]
CcMYB330	Activator	*PAL*	ACCAACAACCAAA	*CcMYB330* silencing reduces capsaicin accumulation in chilli pepper fruits	[133]
CaMYB31	Activator	*AT3*	AAACCA	*CcMYB31* silencing reduces capsaicin and dihydrocapsaicin contents	[134]
CaMYB31	Activator	*4H*, *COMT*, *KAS*, *pAMT*, *AT3*	TAACAAA, [AG]GATT	*CaMYB31* silencing reduced the expression of capsaicinoid biosynthetic genes and the capsaicinoid content	[98]
CaMYB37	Activator	*AT3*	AAACCA	*CcMYB31* silencing reduces capsaicin and dihydrocapsaicin contents	[135]
CcMYB48	Activator	*AT3*, *KAS*	-	*CaMYB48* silencing causes the capsaicin and dihydrocapsaicin contents to decrease by 43% and 45%, respectively	[88]
CaMYB108	Activator	*COMT*, *AMT*, *BCAT*, *FatA*, *BCKDH*	*-*	*CaMYB108* silencing leads to the content of dihydrocapsaicin and capsaicin decreasing 45%	[136]
CcbHLH68	Activator	*CcCOMT*	-	*CcbHLH68* silencing leads to the content of capsaicinoids decreasing by 40.9%	[137]
CcERF2	Activator	*PAL*, *4H*, *4CL*, *COMT*, *pAm*	-	*CcERF2* silencing leads to the content of capsaicin and dihydrocapsaicin being reduced by 74.2 and 73.0%, respectively	[100]

## Data Availability

The original contributions presented in this study are included in the article. Further inquiries can be directed to the corresponding authors.

## Abbreviations

*ACS*	*Acyl-CoA synthetase*
*AT*	*Acyl-transferase*
*HCT*	*Hydroxycinnamoyl transferase*
*C3H*	*p-coumarate 3-hydroxylase*
*C4H*	*Cinnamatc 4-hydroxylase*
*COMT*	*Caffeic acid O-methyltransferase*
*CHS*	*Chalcone synthase*
*DHAD*	*Dihydroxyacid dehydratase*
*HSS*	*Homospermidine synthase*
*PAD*	*Parental advice-1*
*PAL*	*Phenylalanine ammonia-lyase*
*PAT*	*Prephenate aminotransferase*
*PAMT*	*Putative aminotransferase*
PEG	polyethylene glycol
*TD*	*Thr deaminase*
*4CL*	*4-Coumarate-CoA ligase*
*MLO2*	*Mildew locus O2*
